# Reducing anxiety symptoms in adolescents with pre-existing depression: results from a randomized control trial

**DOI:** 10.3389/fpsyt.2025.1461887

**Published:** 2025-06-19

**Authors:** Henriette Solberg, Thormod Idsoe, Serap Keles

**Affiliations:** ^1^ Faculty of Educational Sciences, Department of Special Needs Education, University of Oslo, Oslo, Norway; ^2^ Knowledge Centre for Education, University of Stavanger, Stavanger, Norway

**Keywords:** depression, anxiety, group CBT, adolescents, randomized control trial

## Abstract

**Introduction:**

Depression and anxiety have a long history of co-occurrence, with a relatively high prevalence in the Norwegian population both separate and combined. In adolescence, this prevalence increases significantly and may impact youths’ social and academic functioning drastically. Having effective treatments aimed at adolescents may have potential to prevent both short and long-term effects associated with these disorders. The aim of the current study was to examine whether the “Adolescent Coping with Depression Course” (ACDC), a cognitive behaviour therapy (CBT)-based group intervention aimed at adolescents with subclinical mild-to-moderate depressive symptoms, would also be effective in reducing anxiety symptoms.

**Methods:**

Data, which came from a two-arm parallel cluster randomised control trial conducted in Norway, were collected from 228 adolescents, 133 of whom were assigned to the 14-week ACDC intervention and 95 were assigned to the usual care condition. The data were analysed with structural equation modelling

**Results:**

The results indicated that the intervention is effective in reducing anxiety symptoms via reducing the depressive symptoms, thus illustrating an indirect effect.

**Discussion:**

The findings suggested that ACDC has the potential to reduce anxiety symptoms over time through its effect on depressive symptoms. Implications of the results are presented.

**Clinical trial registration:**

https://www.isrctn.com, identifier ISRCTN19700389.

## Introduction

Many individuals who are diagnosed with either depression or anxiety, also meet the criteria for diagnosis or exhibit a considerable number of symptoms for the other diagnosis (1–3). The co-occurrence and phenomenological overlap between these conditions suggests that effective treatments for one may have secondary benefit for the reduction of symptoms in the other. The aim of the present study was to investigate whether this was the case for a group-based treatment for depressed adolescents (4, 5).

Depression and anxiety are deemed common mental health disorders, and the numbers globally seem to be on the rise (6). In Norway, it has been estimated that somewhere between every fourth and fifth person will struggle with symptoms of anxiety at some point over the course of their life (7); the same seems to be accurate for depression (8). Around 10% of the adult population will develop a depressive disorder over the course of a year, while close to 15% will develop an anxiety disorder (8). This development seems to be mirrored in adolescence, where mental health problems also seem to be on a steady rise (9).

Though relatively low in childhood, the prevalence of both disorders reaches around 20% in late adolescence (10, 11). Anxiety disorders tend to have an earlier onset than depression, as certain types, for example separation anxiety, develops in early childhood (10). In Norway it is assumed that around 5% of all citizens under the age of 18 receive a specialized treatment for their mental health problems (12). Anxiety and depression can have several consequences for those affected, including underperformance in school, higher dropout rates and problems maintaining meaningful social relationships with peers (13, 14); this may further affect their adult functioning in terms of career choices and income possibilities (15). When both disorders are present, they appear to have more severe consequences and to be more difficult to treat (2). Potential extensive functional impairments may warrant addressing symptoms prior to transition to diagnosis, suggesting that finding effective low-level treatments for them is important to avoid symptoms developing into disorders.

Depressive and anxiety disorders in adolescents are typically diagnosed based on criteria outlined in the Diagnostic and Statistical Manual of Mental Disorders (DSM-5; 16). Major Depressive Disorder (MDD) is characterized by the presence of at least five symptoms over a two-week period, including persistent sadness or irritability, loss of interest or pleasure, significant changes in appetite or sleep, fatigue, difficulty concentrating, feelings of worthlessness, and suicidal thoughts or behaviors (16). Generalized Anxiety Disorder (GAD), one of the most common anxiety disorders in adolescents, is diagnosed when excessive worry occurs on most days for at least six months, accompanied by symptoms such as restlessness, fatigue, difficulty concentrating, irritability, muscle tension, and sleep disturbances (16). Other anxiety disorders, such as Social Anxiety Disorder and Panic Disorder, have distinct criteria but share common features of excessive fear and avoidance behaviors (17). Given the high prevalence and comorbidity of these disorders in adolescents, early interventions targeting symptom reduction may help mitigate long-term negative outcomes.

The comorbidity between anxiety and depression can, however, be complicated to measure; in their review of prevalence of common mental health disorders, the World Health Organization refrained from commenting on it (6). Perhaps this is not surprising; the level of comorbidity reported in scientific publications vary greatly (1). This could be due to the fact that the two can be difficult to differentiate because symptoms and consequences may appear relatively similar (13, 18, 19). Though the core symptoms, a persistent feeling of sadness and excessive fear and anxiety, differ, both disorders may lead to avoidance behavior, low self-esteem and affect cognitive functioning (10, 18). Consequences of both disorders in adolescence may include lower performance in school, issues with obtaining and maintaining social relationships with peers and it may lead to avoidance of both school activities and social situations due to a lack of self-confidence (13, 19).

Depression and anxiety share considerable symptom overlap and high comorbidity, raising nosological questions regarding their classification as distinct disorders (20, 21). Ontologically, both conditions may be better conceptualized not as discrete entities but as points along a shared internalizing spectrum, as suggested by frameworks such as the Hierarchical Taxonomy of Psychopathology (HiTOP; 22). Understanding depression and anxiety through a dimensional, transdiagnostic lens may better capture the complex, intertwined nature of adolescent psychopathology.

The theoretical frameworks for understanding depression and anxiety also display similarities. Depression and anxiety in adolescents are influenced by multiple interacting factors, which can be understood through biological (e.g., genetic predisposition, neurochemical imbalances), psychological (e.g., dysfunctional thought patterns, negative cognitive biases), and psychosocial (e.g., the impact of environmental stressors, adverse childhood experiences, and social relationships on mental health) models. For example, according to Beck’s cognitive theory (23), depression can be understood through dysfunctional information processing (24). People with symptoms of depression tend to have negative thoughts and feelings about themselves and their abilities to function in social and academic settings. These thoughts affect how they interact with the world around them and how they interpret and evaluate their success in these interactions, creating a more persistent tendency of negative thinking and emotional distress (25). One of the basic components of Beck’s theory is the idea of schemas, which describes how these thoughts are automated and disturbs the possibility of gaining new and more positive experiences (26). These ideas can also be applied to anxiety. In fact, Clark and Wells (27) have exemplified this with their own cognitive model for understanding social anxiety, which includes many of the same elements (14). Anxiety is explained as an introspective process in which the person affected interprets the situation they find themselves in based on stress and worry previously experienced in a similar situation. Based on the stress experienced and the perceived negative reactions from their surroundings, situations may wrongfully be deemed a failure and amplify the negative automatic thoughts associated with social situations. Thus, both symptoms of depression and anxiety can be understood through dysfunctional information processing and a learned negative perception that is difficult to break out of.

## The current study

Treatment for anxiety and depression often share common traits, and both medication and treatment plans with therapists may be similar in structure (10, 11, 28). For example, cognitive behavioral therapy (CBT) is the most commonly used treatment form for depression in adolescents (29), as well as the most commonly used treatment form for anxiety (1). Given the prevalence of both disorders among adolescents, both combined and separate, having an intervention that successfully reduces symptoms of both would be beneficial. Effective early intervention could limit the development of symptoms and the consequences they have for the individual. AUTHOR CITE (2016; 2019; 2020) have conducted a study on the Adolescent Coping with Depression Course (ACDC), a CBT group intervention for adolescents with mild to moderate type depression.

Several CBT-based group interventions for adolescents exist, such as the ‘Coping with Depression Course’ (CWDA) (30). Even though CWDA share many similarities with ACDC, the latter has some interesting additions by offering a more integrative and transdiagnostic approach that targets core maladaptive cognitions underlying both depression and anxiety (29). In addition to classic CBT techniques like structured modules focusing on cognitive restructuring (identifying and challenging negative automatic thoughts), behavioral activation (increasing engagement in rewarding activities), emotional regulation strategies, and skills for improving interpersonal relationships, ACDC, on the contrary from CWDA, integrates more components from other perspectives. These are elements from Rational Emotive Behaviour Therapy (REBT) (31) to address maladaptive beliefs. Aspects of Wells’ Meta-Cognitive Therapy (32) targeted worry and rumination. Meta-Cognitive Theory adds to CBT by focusing on how we reflect on our style of thinking, in addition to the thinking style as described in CBT. By so doing, ACDC merges the two perspectives in ways that allow for dealing with how quality and style of thoughts may change, and thereby hopefully improve dysfunctional thoughts in a better way. Positive psychology exercises (33) emphasized building strengths and cultivating optimism. Even though effects of positive psychology are less documented for adolescents, ACDC combines it with the other theories described here, e.g. by emphasizing awareness and training in breaking thought patterns. Neurobiological perspectives are used to embrace the other perspectives to give better understandings of information processing and why people react in ways they do, sometimes automatically. Homework assignments reinforced skills between sessions (AUTHOR CITE).

It was found that the primary study (ACDC) had a mild to moderate reductive effect (effect size = -.31) on depressive symptoms in the intervention period (AUTHOR CITE). Reduction was kept up at 6-and 12-months after the intervention period had ended (AUTHOR CITE). Considering the comorbidity of depressive and anxiety symptoms, we examined whether this particular intervention could also be effective in reducing symptoms for participants’ comorbid anxiety.

The aim of the current study was to explore whether the ACDC intervention could reduce anxiety symptoms directly, or whether it reduced depressive symptoms that again could lead to a reduction in anxiety symptoms. In order to investigate this, we explored two questions. First, we investigated whether the intervention has a direct effect on reducing anxiety symptoms. Second, we examined whether the intervention has an indirect effect through the reduction of depressive symptoms. This may be expressed in the two following research questions:

1) Will the ACDC intervention developed to reduce depressive symptoms have a direct effect on comorbid anxiety symptoms among depressed adolescents?
2) Will the ACDC intervention have an indirect effect on comorbid anxiety symptoms through the reduction of depressive symptoms?

It is important to note that our study focuses on depressive and anxiety symptoms rather than clinically diagnosed depressive and anxiety disorders. Symptoms of these conditions can be present at varying levels of severity without meeting full diagnostic criteria, and targeting symptom reduction through early interventions may help prevent the progression to clinical disorders.

## Method

The data was collected through the Adolescent Coping with Depression Course (ACDC) study. The study was designed as a two-arm parallel cluster randomized controlled trial (4, 34). The project had a pre-post-follow-up longitudinal design, as data was collected about the effects of the intervention over an extended period of time, also after the intervention period ended.

### Ethical considerations

At the initial screening, all participants were presented with information about the purpose and procedure of the study (4). They received information about both study conditions, the intervention group and the control group. The participants were also informed that the information collected would be stored in a safe manner by the researchers. They were then told that they would be allowed to withdraw from the study at any point. This is all in accordance with the principle of informed consent. The project was approved by the Norwegian Regional Committee for Medical and Health Research Ethics (South East).

### Participants

Course leaders were recruited from school mental health services, the Educational Psychological Counselling Services (PPT) and the Norwegian public mental health service for children and adolescents (BUP) (4, 5, 34). These course leaders were then randomly assigned to either experimental (N=18) or control (N=17) conditions by the administrative personnel from the Norwegian Centre for Child Behavioral Development (NUBU). Course leaders assigned to the intervention group received a full week of training as specified in the ACDC protocol before the recruitment of the participants and the intervention periods started. Leaders of the “usual care” (UC) control groups received a one-day course in how to recruit participants. They were offered training in the ACDC intervention shortly after the intervention period had ended, so that they could benefit from participation without affecting the results of the study.

Youth participants were recruited directly by course leaders through various channels available to them (4, 5, 34). This could either be by placing information in schools and health care centers, through general practitioners, or advertisements in local newspapers. Participants were recruited either through direct contact with the course leaders or by referral from general practitioners, psychiatric clinics and school services. The target demographic was upper secondary school students, those who were 16 and 17 years old. (Even though the ACDC is described as being aimed at adolescents aged 14 to 20 years, the reason for the age range was that the funding of our study was assigned to investigate dropout from upper secondary school, limiting the age range to 16–17 years old). Based on evaluations from clinical interviews they had to have subclinical depression or mild to moderate depression in accordance with the DSM-IV-TR criteria (35) supported by cutoff criteria from the Beck Depression Inventory (BDI) (36) (see further down). Exclusion criteria included a presence of bipolar disorder, psychosis, substance-use, ADHD and brain damage (4, 37).

Potential participants were recruited by course leaders without knowing what condition they were being assigned to (4, 5, 34). In other words, they received information about both conditions and signed a consent form consenting to whichever condition they would receive. This was important in order to reduce post-randomization selection bias (4). In their first meeting with the leaders, the adolescents were screened using the Beck Depression Inventory (BDI) and had a brief clinical interview to determine whether they met the inclusion criteria for the study. According to the guidelines for BDI, those who have a score ≥ 10 will satisfy the cutoff criterion for mild to moderate depression (36). This was one of the main inclusion criteria evaluated during the clinical interview. If the adolescents satisfied this criterion, and were 16 or 17 years old, as well as not excluded according to the criteria mentioned above, the adolescents could be included in the RCT.

**Figure 1:**
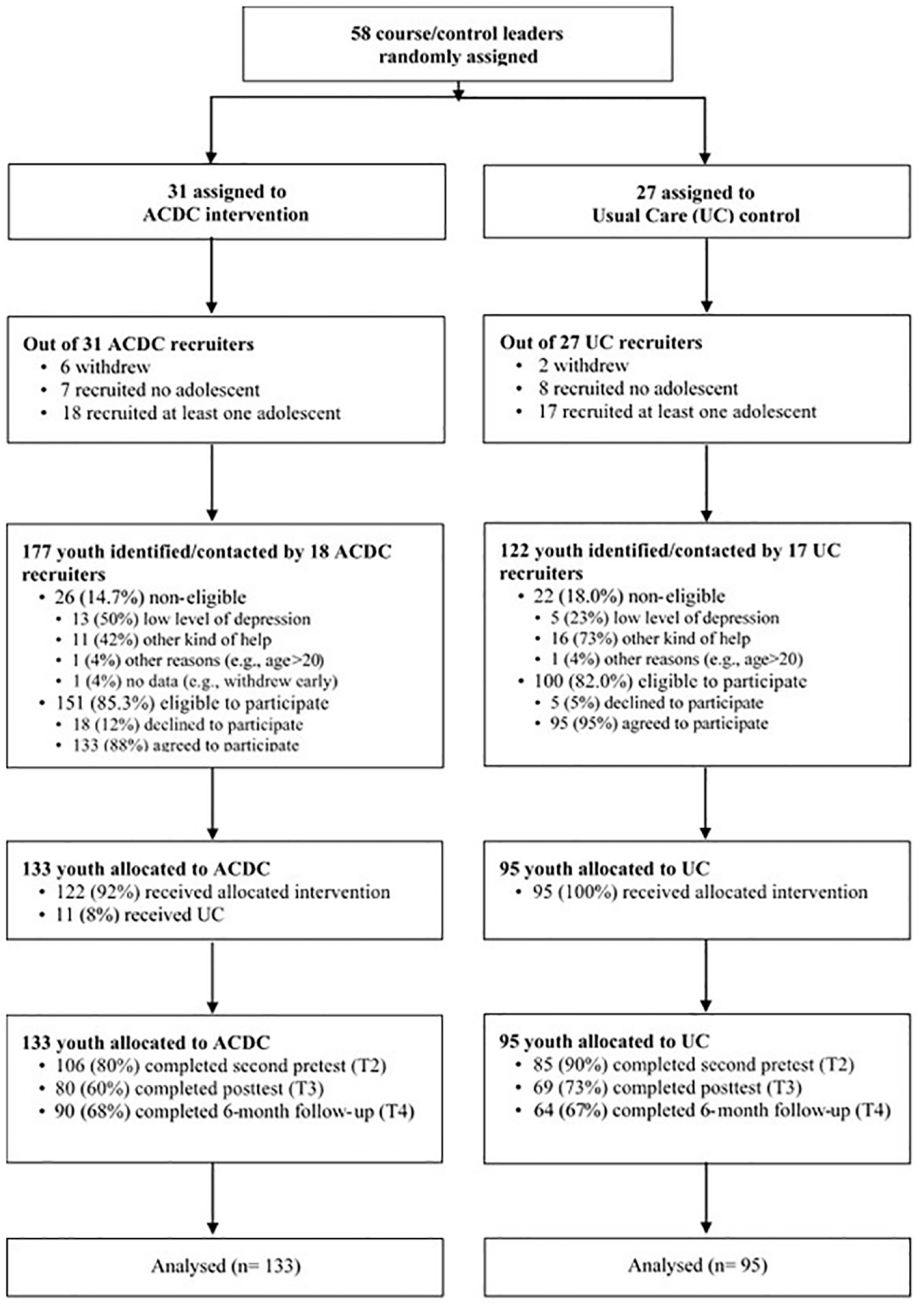
Participant flow chart for the sample in the ACDC study.

The 18 ACDC recruiters contacted 177 adolescents. 26 of them were non-eligible according to the inclusion and exclusion criteria. Of the 151 remaining adolescents that were eligible, 133 agreed to participate. The 17 recruiters of usual care came in contact with 122 adolescents that were invited for the clinical interview. 22 of them (18%) were non-eligible according to the inclusion and exclusion criteria. 95 of the remaining eligible 100 adolescents agreed to participate in the study. In total, 228 participants (88% girls, mean age = 16.70, *SD* =1.14) were eligible participants following screening, with 133 participants in the ACDC intervention group and 95 in the UC control group (see **Figure 1**).

### Measures

#### Anxiety symptoms

A modified version of items measuring anxiety from the HSCL-10 (Hopkins Symptom Checklist) was used to assess anxiety symptoms. The participants were asked to think about each symptom and then rate their agreement on a 4-point Likert scale (*1* = strongly disagree to *4* = strongly agree). An example statement would be ‘I suddenly get scared for no reason’. High scores on the scale indicated higher levels of anxiety. Cronbach’s alpha for the total symptom scale in this current study ranged between .844 and .889 across waves.

#### Depressive symptoms

While BDI was used for eligibility, the Center for Epidemiologic Studies’ Depression Scale for Adolescents (CES-D) was used for depressive symptom outcome measure. This is a short self-report measure designed for use in population studies (38). It consists of 20 items, based on common symptoms of depression identified through research literature. These components include depressed mood, feelings of guilt and worthlessness, helplessness and hopelessness, psychomotor retardation, and effect on appetite and sleep disturbance (38, p. 386). The participant is presented with a set of statements and asked to rate these on a four point Likert scale, with a total possible score of 0–60 points. Higher scores indicated the presence of higher level of depressive symptoms. Cronbach’s alphas for the symptom scale ranged between 881 and 921 across waves.

#### Demographic variables

Gender and age for the participants were collected at the screening interviews.

### Intervention versus control groups

#### Intervention group: the “Adolescent Coping with Depression Course”

The Adolescent Coping with Depression Course (ACDC) is a CBT-based group intervention aimed at adolescents aged 14 to 20 years old with mild to moderate symptoms of depression (4, 5, 29, 34, 39). The intervention aims to reduce symptoms of depression before they worsen into a diagnoseable disorder. This is done in accordance with the philosophy of CBT, which is that depression might be caused by dysfunctional thinking, and that it can be dealt with by changing the negative thought patterns that cause the individual distress (29).

Throughout the course, the participants are taught ways to monitor and analyze their own perceptions, by challenging the notions about themselves and their worldview that are destructive and causes stress and negative feelings. The course is delivered in a group format over 8 weekly sessions of approximately 2 hours. Two follow-up sessions to the programme are conducted 3 and 6 weeks after the end of the programme, which makes for a total of 10 sessions. ACDC is mainly built on the principles from Beck’s cognitive behavior therapy (CBT) and Ellis’ Rational Emotive Behaviour Therapy (REBT), but it also includes principles from Wells’ Meta-Cognitive Theory and Seligman’s Positive Psychology (4). The implementation of the intervention followed a standardized manual, and the sessions were structured according to the following topics: 1) How feelings arise, 2) Situations and thoughts that can lead to sadness, 3) Regulating feelings with thoughts, 4) Regulating feelings with actions, 5) Thinking in better ways, 6) Training in unaccustomed thoughts: Positive thinking and contact, 7) Better contact with others, 8) Coping and cognitive techniques, 9) Daily use of methods. Work and practice, 10) Daily use of methods. 11) Conclusion.

Based on the clinical evaluation and supported by BDI criteria, adolescents presenting with severe depressive symptoms were excluded. In addition, exclusion criteria also included a diagnosis of bipolar disorder, psychotic disorders, substance-use disorders, ADHD, or brain injury.

#### Control group: usual care

The participants in the control group received usual care, i.e., the treatment the control group leader would typically provide for this group at their workplace (4, 5, 34). This could involve referral to an array of different care providers, like psychologists, doctors, school nurses or teacher, who may provide a wide array of treatments, like conversations, various standard treatments, the use of pharmacotherapy or no treatment. There were no restrictions as to what type of treatment the control group participants could receive. The participants in this group were asked to report how they were referred and who they received care from.

### Procedure

The data used in this study was collected through a self-report questionnaire filled out by the participants at different time points (4, 5). The first time point (T1) was a pre-test where data was collected about both depression and anxiety, as well as other potential mediators and moderators as described in Idsoe et al. (4). This data was collected at the screening interviews for the study. In addition, depression was measured a second time just before the intervention period started (T2). Both depression and anxiety were measured directly after the intervention period ended (T3) and as part of a follow up questionnaire delivered 6 months after the intervention ended (T4). The questionnaire was delivered to all participants digitally at all time points, apart from at the initial screening (T1), where participants answered on paper.

### Statistical analyses

The analysis was based on the *intention-to-treat* (ITT) principle, which aims to preserve the validity of a randomized control trial by keeping the randomization while simultaneously ensuring that the groups maintain comparability (40, 41). An ITT analysis will seek to analyze all participants in a trial in the group context which they were assigned to, regardless of whether they received any treatment or not (40). Because it preserves randomization, the ITT approach is deemed the preferred method of analysis when conducting a randomized control trial (42).

SPSS21 was used for descriptive statistics. Mplus8 was used to test the hypothesized models with repeated data points. For the first model, we tested whether the intervention had a direct effect on post–test anxiety symptoms, after controlling for demographic variables such as gender and pre–test anxiety symptoms. For the second model, we tested whether the intervention had an indirect effect on the 6–month follow–up anxiety symptoms through its effect on the post–test depressive symptoms, controlling for demographic variables and pre–test depressive and anxiety symptoms. To reduce the effects missing data could have on the results as well as to accommodate for non–normal item distribution, the robust maximum likelihood (MLR) estimator was used in MPlus. A full information maximum likelihood (FIML) procedure, which is in accordance with ITT and uses all data points, was employed. Pre–test variables associated with missingness were used in the models as either control variables or auxiliary variables (4, p. 8). The ITT analyses included auxiliary variables based on the assumption of missingness to be at random (MAR), after investigating the missing data patterns. In addition to chi-square tests, other fit indices were used and reported: the root-mean-square error of approximation (RMSEA), the comparative fit index (CFI), the non-normed fit index (Tucker Lewis Index-TLI), and the standardized root-mean-square residual (SRMR). Hooper et al. (43) recommends that RMSEA <.070, CFI >.95, TLI >.90 and SRMR <.080 represents a well-fitting model.

## Results

### Descriptive statistics

**Table 1** shows the descriptive statistics for the variables examined with the mean and standard deviation for both the intervention group and the control group, and **Table 2** exhibits the correlations among the study variables.

**Table 1:** Descriptive statistics of the measures for the intervention and control group in the Adolescence Coping with Depression Course (ACDC) study.

	ACDC intervention (N = 133)		UC control (N = 95)	
	*M*	*SD*	*M*	*SD*
1. Depression T1-pretest	33.08	9.97	32.01	9.75
2. Depression T2-2^nd^ pretest	32.77	8.80	30.28	10.67
3. Depression T3-post test	26.85	11.82	29.55	10.77
4. Anxiety T1-pretest	2.42	.67	2.36	.64
5. Anxiety T3-post test	2.27	.77	2.37	.72
6. Anxiety T4-6m follow up	2.28	.74	2.24	.63
7. Gender (%)	91.0		83.2	
8. Age	16.55	1.10	16.92	1.16

Ranges and anchors: Depressive symptoms (0 = No symptoms, 60 = High level of and frequent symptoms); Anxiety symptoms (1 = Strongly disagree, 4 = Strongly agree).

**Table 2:** Intercorrelations for the variables of the overall sample (N=228).

	1	2	3	4	5	6	7	8
1. Depression T1-pretest	(.881)	.65**	.43**	.59**	.45**	.36**	.21**	-.07
2. Depression T2-2^nd^ pretest		(.881)	.47**	.47**	.44**	.44**	.13	-.16*
3. Depression T3-post test			(.921)	.38**	.70**	.52**	.07	.00
4. Anxiety T1-pretest				(.844)	.52**	.44**	.22**	.05
5. Anxiety T3-post test					(.889)	.56**	.09	-.02
6. Anxiety T4-6m follow up						(.857)	-.09	-.08
7. Gender							(-)	-.16*
8. Age								(-)

*p < 0.05, **p < 0.01. Gender: Males are coded as 1 and females as 2. Cronbach’s Alpha are given in parentheses.

Anxiety and depression were significantly and positively correlated across all time points, indicating that higher scores of depression correlated with higher scores of anxiety. Gender correlated with both anxiety and depression at T1 (*r* = .214, *p* < 0.01 and *r* = .22, *p* < 0.01, respectively), indicating that girls scored slightly higher on both of these variables prior to the intervention.

### Structural equation modelling

**Figure 2:**
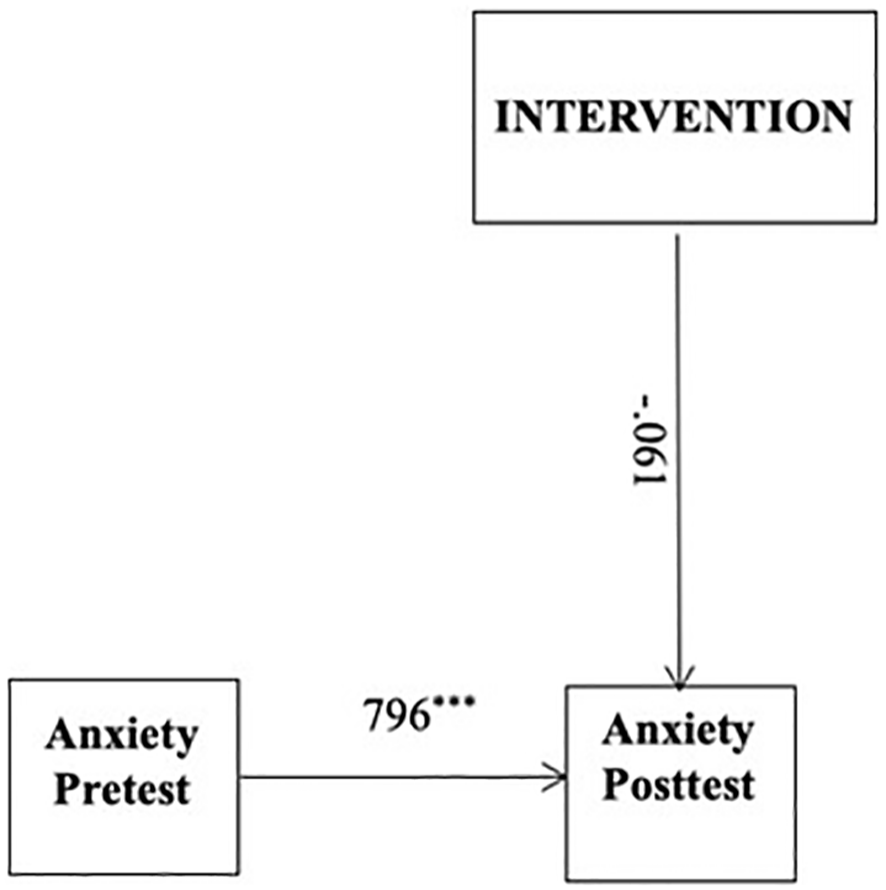
Direct model. Intervention: UC coded as 0 and ACDC coded as 1. Standardised parameter estimates were reported. ***p <.001.

In line with the research questions, two models were tested. The results of the model hypothesizing the direct effect of the ACDC intervention on anxiety symptoms were illustrated in **Figure 2**. Gender and age were controlled for, but dropped from further analyses because they were not significantly associated with the other variables. The anxiety post-test variable (T3) was regressed on the intervention variable. The diagram thus shows the direct effect of the intervention on anxiety symptoms. The fit indices of the proposed model were RMSEA = .073, 90% CI [.033,.112]; CFI = .957; TLI = .922; SRMR = .061. The results revealed that the group receiving the ACDC intervention did not have a significantly greater decrease in anxiety symptoms than those who received UC (*β* = -0.61, *p = .*667). This reveals that the ACDC intervention did not have a direct effect on anxiety symptoms.

**Figure 3:**
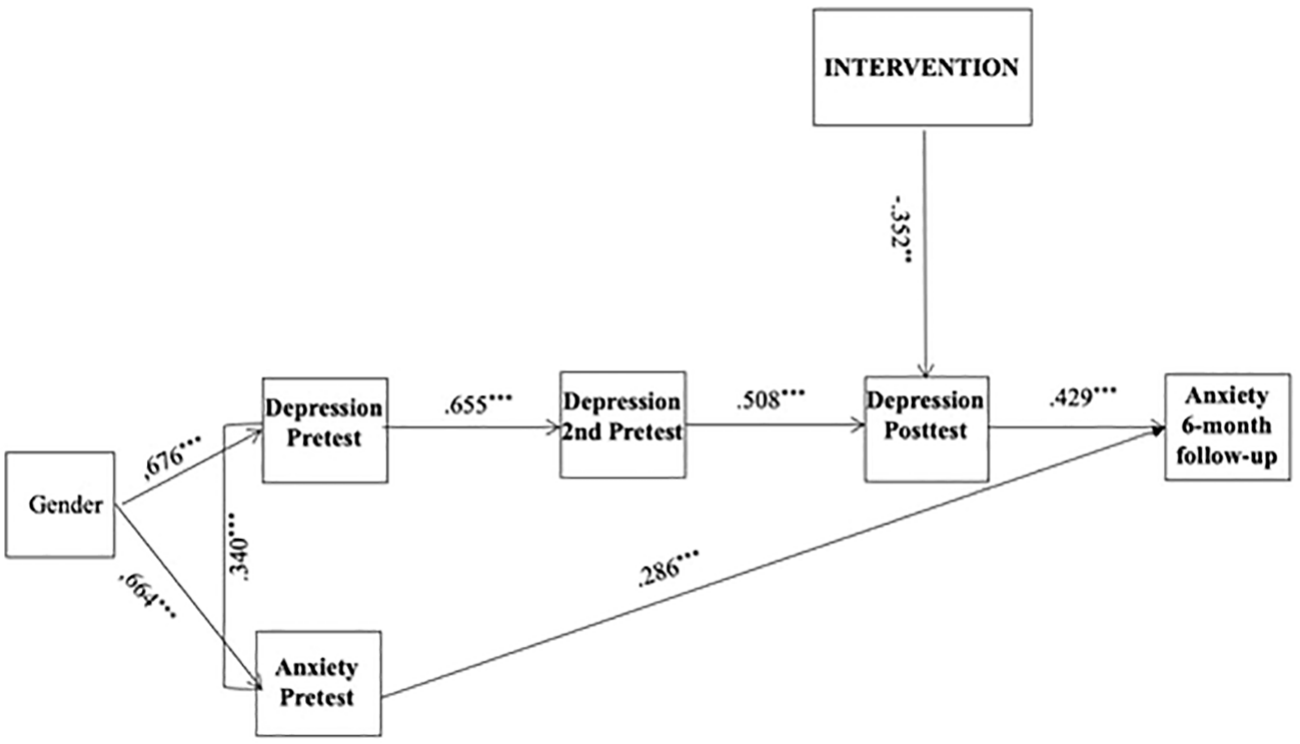
Indirect model. Gender = Males coded as 1 and females coded as 2. Intervention groups = UC was coded as 0 and ACDC as 1. Standardised parameter estimates are reported. **p <.01 ***p <.001.

The results of the indirect model were displayed in **Figure 3**. Baseline depressive and anxiety symptoms were controlled for in the analysis. Gender and age were also controlled for in the initial model, but age was dropped from further analyses because it was not significantly associated with any of the variables. In line with the indirect model, the post-test depressive symptoms were regressed on the intervention variable, after controlling for pre-test depressive and anxiety symptoms. The 6-month follow-up anxiety symptoms were regressed on the post intervention depressive symptoms. The path diagram shows a longitudinal effect of ACDC on anxiety symptoms via reducing depression measured at post-test. The fit indices of the model had an acceptable model fit, RMSEA = .73, 90% CI [.033,.112]; CFI = .957; TLI = .922; SRMR = .061. In line with the second hypothesis, the results revealed that the symptoms of depression had a greater decrease in the group who received the ACDC intervention (*β* = -.352, *p* <.01). In line with the second hypothesis, depressive symptoms at post-test positively predicted anxiety scores at the 6-month follow-up (*β* = .429, *p* <.001). Furthermore, ACDC, which resulted in a larger decrease in depressive symptoms at post-test, had an indirect effect on adolescents’ anxiety symptoms at 6-month follow up (indirect *β* = .151, *p* = .019).

## Discussion

The aim of this study was to examine whether the ACDC intervention, which is a CBT-based course for depressed youth, had the potential to reduce anxiety symptoms in adolescents with concurrent depressive symptoms. In order to investigate this, we tested two models: 1) whether the ACDC intervention had a direct effect on reducing anxiety symptoms, and 2) whether the intervention had an indirect effect, i.e. reducing anxiety symptoms through the reduction of depressive symptoms. The results of the study suggested that ACDC has the potential to reduce anxiety symptoms over time through its effect on depressive symptoms.

Even though there was no direct effect of ACDC om anxiety symptoms, the findings support previous studies that found CBT to be an effective treatment for anxiety (44). This can be due to a multitude of reasons. CBT for anxiety typically include some form of exposure therapy, to help the patient face and overcome the situations that causes them stress (2, 45). This is not a part of the ACDC intervention, which might be why the intervention fails to have a direct effect on anxiety symptoms themselves. More knowledge on how to build an effective CBT programme also for anxiety symptoms could enable us to adapt the ACDC intervention so that we could more successfully target anxiety behavior. This could in the future contribute to ACDC becoming an effective treatment for both symptom groups with small adjustments.

Much of the theoretical framework of the ACDC intervention comes from Beck’s cognitive theory, where depression is understood through an internalized negative understanding of oneself and one’s abilities in interaction with the surroundings (24). As previously stated, this theory or similar theories and models can and have been used to explain mechanisms related to anxiety (14). Depression and anxiety also tend to share many symptoms (10, 11, 13, 28) and have similar consequences in adolescence (13, 19). These similarities in understanding, symptoms and consequences could suggest that the ACDC intervention targets certain underlying factors that symptoms of anxiety and depression have in common. This could explain the indirect effect on anxiety symptoms. Going forward, a better understanding of the relationship between depressive and anxiety symptoms could lead to a better understanding of how to develop effective interventions that successfully target both symptom groups.

Anxiety is not a uniform concept; rather, it is a set of thoughts and behaviors that can be used to describe many different issues where fear and worry are the core symptoms (10). Common anxiety disorders are separation anxiety, social anxiety disorder (SAD), specific phobia, panic disorder, generalized anxiety disorder (GAD) and post-traumatic stress disorder (19). Differentiation of anxiety disorders is mainly reliant on understanding the causes behind the behavior. The ACDC project measured general anxiety symptoms (34). Therefore, we cannot say whether the ACDC intervention would be more effective for any one of these disorders. Based on demographic characteristics in our sample, we can suspect that it would be effective for people who experience symptoms of SAD or GAD, as those can be considered two of the most common diagnosis for our target age group (46). This should be tested in future studies. In addition, another important consideration is whether some participants may have met criteria for Mixed Anxiety-Depressive Disorder (MADD). While our study focused on depressive and anxiety symptoms rather than formal diagnoses, we did not specifically rule out MADD. Future research should consider differentiating between general symptomatology and specific diagnostic categories to refine intervention targeting.

It is also possible that for some participants, anxiety symptoms remitted as part of the natural recovery from a depressive episode, independent of the intervention effects. Given the well-documented relationship between depression and anxiety, symptom fluctuations may occur even without targeted treatment. Future studies should incorporate additional control conditions or longitudinal designs to better isolate intervention effects.

Lastly, another potential confounding factor is the role of external stressors. Anxiety symptoms may have changed in some participants due to the effects of external stressors. While we did not specifically assess changes in life stressors that may have contributed to anxiety symptom fluctuations, future research should include measures of stressful life events to account for their potential impact on symptom trajectories.

### Study limitations

There are certain limitations to this study that might be considered while interpreting the results. The ACDC data might be vulnerable to both over- and underreporting of symptoms, as it relies on self-reported measures of the participants’ symptoms. A prominent limitation in our sample is also the gender imbalance. With 88% girls, it is clear that boys were underrepresented in this sample. This could illustrate that boys are less likely to seek help for their mental health issues (4). The gender imbalance also mirrors a tendency found in a Norwegian study during the covid-19 pandemic, where the amount of adolescent girls seeking help for their mental health increased, while it was stable for boys (9). Though studies typically find that girls are twice as likely to suffer from both depression and anxiety (10, 11), there is not enough evidence for us to say something about how this intervention affect boys. More research would be needed in order to discuss the effects of this intervention for boys. Including more boys in the sample would also increase the statistical power to run multiple group analyses to see whether gender might moderate potential effects on anxiety. Other potential mediators/moderators that should be investigated according to cognitive theories are negative automatic thoughts, dysfunctional attitudes, and rumination.

Six participants (8.2%) in the intervention group and eight participants (11.9%) in the control group reported receiving pharmacotherapy during the intervention period. Among those in the intervention group, four indicated using anti-depressives, one reported using anxiolytic and one reported using “both”. In the control group, four participants reported using anti-depressives, while two reported both anti-depressives and anxiolytics and two did not know. Overall, the use of medication was comparable between the two groups.

Attrition can affect the interpretation of the results of a study, by altering the composition of the sample that is supposed to represent the population (47). Longitudinal studies are especially vulnerable to the effects of attrition. Attrition did occur in this study, but the researchers made a significant effort to handle it through the design and trial period. Several steps were taken to reduce bias. First of all, no significant baseline differences in depression were found between the attrition group and those who continued for post-test. After approval from the Norwegian Regional Committee for Medical and Health Research Ethics, we also contacted the attrition group and asked their reasons for not continuing. Most of the reasons given had a practical nature, and we found no specific differences in reported reasons across treatment and control conditions. Several other variables predicted attrition, and by adding them as auxiliary information to our models we also reduced bias in accordance with modern missing data approaches (48).

Group CBT provides therapeutic benefits such as normalization of experiences, enhancing social and relational aspects (49), and is more time- and cost-effective than individual therapy (50). However, it can be less flexible due to such factors as reduced individual attention, limited ability to tailor treatment, difficulties in accommodating participants’ needs, interpersonal conflicts within the group, and may pose challenges for adolescents with severe social anxiety (51).

A limitation of ACDC when it comes to potential effects on comorbid anxiety symptoms is the lack of explicit exposure techniques for anxiety, which could potentially enhance direct anxiety symptom reduction.

Finally, it should be mentioned that shared neurobiology may be an important factor in symptom overlap.

### Implication for future research

Further research on the ACDC intervention and how it can be adapted to better treat symptoms often associated with depression, like anxiety, could make the intervention more versatile. To achieve this, more knowledge about how anxiety and depression interact and affect each other would be needed. A more systematic study on how ACDC affects different types of anxiety and their symptoms could also be beneficial, combined with adaptation of the program using the existing knowledge about anxiety specific treatment mechanisms in order to target anxiety symptoms in a more direct way.

## Data Availability

The raw data supporting the conclusions of this article will be made available by the authors, without undue reservation.
